# Draft Genome Sequence of *Escherichia coli* Strain ATCC 23502 (Serovar O5:K4:H4)

**DOI:** 10.1128/genomeA.00046-13

**Published:** 2013-02-28

**Authors:** Brady F. Cress, Zachary R. Greene, Robert J. Linhardt, Mattheos A. G. Koffas

**Affiliations:** a Department of Chemical and Biological Engineering, Center for Biotechnology and Interdisciplinary Studies, Rensselaer Polytechnic Institute, Troy, New York, USA; b Department of Chemistry, Chemical Biology, Center for Biotechnology and Interdisciplinary Studies, Rensselaer Polytechnic Institute, Troy, New York, USA; c Department of Biology, Center for Biotechnology and Interdisciplinary Studies, Rensselaer Polytechnic Institute, Troy, New York, USA

## Abstract

We report the 4.682-Mbp high-quality draft assembly of the *Escherichia coli* strain ATCC 23502 (serovar O5:K4:H4, also known as NCDC U1-41) genome. This uropathogenic strain, commonly referred to as *E*. *coli* K4, produces a glycosaminoglycan-like capsular polysaccharide with a backbone similar in structure to unsulfated chondroitin, a precursor to the nutraceutically and potentially pharmaceutically valuable compound chondroitin sulfate. Metabolic reconstruction of this genome will enable prediction of genetic engineering strategies leading to increased chondroitin production.

## GENOME ANNOUNCEMENT

*Escherichia coli* strain ATCC 23502 is a uropathogenic strain that produces a group 2 capsular polysaccharide known as K4 capsular polysaccharide (K4 CPS) (1). The K4 capsular polysaccharide consists of a repeating [→4) β-d-glucuronic acid (GlcA) (1 → 3) *N*-acetyl-β-d-galactosamine (GalNAc) (1→]_*n*_ disaccharide backbone with bisecting β-fructofuranose subunits linked to C3 of GlcA (2); the polysaccharide resembles unsulfated chondroitin sulfate with the exception of the terminal fructose residues present in K4 CPS. Group 2 capsules are of interest to the pharmaceutical industry as precursors in non-animal-sourced glycosaminoglycan (GAG) production, a safer alternative to animal tissue extraction routes that are susceptible to mammalian virus and prion contamination. Chemical or enzymatic sulfation of defructosylated K4 CPS could produce a range of chondroitin sulfate polysaccharides that are already used in the treatment of osteoarthritis and that have shown promise as skin substitutes, antivirals, and vaccines against maternal malaria (3). Genome-scale reconstruction of *E. coli* strain ATCC 23502 metabolism will allow for the prediction of gene deletions and overexpressions capable of increasing chondroitin production.

Genomic DNA was purified from *E. coli* strain ATCC 2352 with an Invitrogen PureLink Genomic DNA mini kit. The genome was sequenced using the Illumina HiSeq 2000 sequencing system, which produced 65 M paired-end reads of 101 bp with an insert size of 400 bp. Approximately 28 M random reads were assembled using Velvet v1.2.07 (4) at an optimal hash length of 93. The final genome assembly has approximately 43-fold coverage and contains 144 supercontigs composed of 160 contigs (>200 bp in length) with a total size of 4,682,525 bp, an N_50_ contig length of 105,137 nucleotides, and a mean G+C content of 50.6%. All assembly data were deposited in the EMBL nucleotide sequence database.

The draft genome was annotated by the RAST (Rapid Annotation using Subsystem Technology) server (5) using Glimmer3 as a gene caller (6), which predicted 4,560 coding sequences (CDSs) with an average length of 894 bp (3,541 CDSs have functional predictions), 83 tRNA-encoding genes, and 25 rRNA-encoding genes. RAST was also used to construct a draft metabolic model (7) containing 1,141 genes, corresponding to 1,389 reactions with 1,098 metabolites (including 4 gap-filling reactions and an artificial biomass reaction). Further analysis of the genome will provide useful information to characterize the entire mechanism of K4 CPS biosynthesis, transport, and extracellular attachment. Reconstruction of this strain’s unique metabolic network will guide metabolic engineering modeling efforts aimed at increasing chondroitin production through targeted genetic manipulations. A comparative genomics analysis between *E. coli* strain ATCC 23502 and other strains producing glycosaminoglycan-like CPS is under way.

### Nucleotide sequence accession numbers.

The annotated draft genome sequence was deposited in DDBJ/EMBL/GenBank under accession no. CAPL00000000. The version described in this paper is the first version, CAPL01000000.
